# p53 and Cell Cycle Dependent Transcription of *kinesin family member 23 (KIF23)* Is Controlled Via a CHR Promoter Element Bound by DREAM and MMB Complexes

**DOI:** 10.1371/journal.pone.0063187

**Published:** 2013-05-01

**Authors:** Martin Fischer, Inga Grundke, Sindy Sohr, Marianne Quaas, Saskia Hoffmann, Arne Knörck, Catalina Gumhold, Karen Rother

**Affiliations:** 1 Molecular Oncology, Medical School, University of Leipzig, Leipzig, Germany; 2 Department of Medical Biochemistry and Molecular Biology, Center of Human and Molecular Biology, Medical Center, Saarland University, Homburg, Germany; Virginia Commonwealth University, United States of America

## Abstract

The microtubule-dependent molecular motor KIF23 (Kinesin family member 23) is one of two components of the centralspindlin complex assembled during late stages of mitosis. Formation of this complex is known as an essential step for cytokinesis. Here, we identified *KIF23* as a new transcriptional target gene of the tumor suppressor protein p53. We showed that p53 reduces expression of *KIF23* on the mRNA as well as the protein level in different cell types. Promoter reporter assays revealed that this repression results from downregulation of *KIF23* promoter activity. CDK inhibitor p21^WAF1/CIP1^ was shown to be necessary to mediate p53-dependent repression. Furthermore, we identified the highly conserved cell cycle genes homology region (CHR) in the *KIF23* promoter to be strictly required for p53-dependent repression as well as for cell cycle-dependent expression of *KIF23*. Cell cycle- and p53-dependent regulation of *KIF23* appeared to be controlled by differential binding of DREAM and MMB complexes to the CHR element. With this study, we describe a new mechanism for transcriptional regulation of *KIF23*. Considering the strongly supporting function of KIF23 in cytokinesis, its p53-dependent repression may contribute to the prevention of uncontrolled cell growth.

## Introduction

Cell division is a fundamental process, which has to be regulated very precisely. This control is achieved by utilizing several mechanisms affecting diverse cell cycle checkpoints. One of the last possibilities to stop cell division is the regulation of cytokinesis. During late anaphase the central region of the microtubule spindle (central spindle) reorganizes to produce the midzone - a tightly packed bundle of antiparallel microtubules [1]. It is known that centralspindlin, a heterotetramer composed of two molecules of Kinesin family member 23 (KIF23) and two molecules of RACGAP1 is critical for this microtubule bundling during cytokinesis [2], [3]. KIF23 is a kinesin-like motor protein which can cross-bridge antiparallel microtubules and drive microtubule movement *in vitro* [4]. It was found to be a nuclear protein that localizes to the interzone of mitotic spindles, acting as a plus-end-directed motor enzyme that moves antiparallel microtubules *in vitro* [5].

In accordance with its essential function in cytokinesis, expression of KIF23 is regulated in a cell cycle-dependent manner. KIF23 expression is repressed in G_0_/G_1_ phase, induced in early S phase and peaks in G_2_/M phase [6]. Genes transcribed differentially during the cell cycle are often controlled through a cell cycle-dependent element (CDE) and/or a cell cycle genes homology region (CHR) in their promoters (reviewed in reference [7]). Interestingly, Seguin *et al.* identified a functional CHR element close to the transcription start site of the *KIF23* promoter and demonstrated that this site is involved in promoter repression of *KIF23* in G_1_ [6]. However, the exact mechanism, particularly the proteins involved, of *KIF23* cell cycle-dependent expression remains to be elucidated.

For a long time, identification of specific CHR binding proteins had been elusive. Recently, experimental data together with bioinformatic analyses suggest that many genes holding phylogenetically conserved CHR elements in their promoters binds a multiprotein complex called DREAM [8]. This complex was first identified in cells of *Drosophila melanogaster* and consists of the *drosophila* Rbf, E2f2/Dp and Myb-interacting proteins mip120, mip130 and mip40. It was determined to be essential for silencing of developmentally regulated genes [9]. Later the corresponding mammalian DREAM complex (DP, Rb-like, E2F4 and MuvB) was discovered [10], [11]. The MuvB core of this complex consists of LIN9, LIN37, LIN52, LIN54 and RBBP4 and is able to recruit E2F4/DP1 or E2F5/DP1 and RB-like proteins p130 or p107. The DREAM complex binds to promoters in G_0_ and early G_1_ and serves to repress transcription [10]. When cells progress through the cell cycle, B-Myb (MYBL2) interacts with the MuvB core and replaces RB-like proteins (p107/RBL1 and p130/RBL2), E2F4 and DP1/2 to form the activating MMB (Myb-MuvB) complex [8], [10]–[14]. Very recently, Müller et al. could demonstrate that both, the DREAM complex and the MMB complex can directly bind to the conserved CHR sites of human and mouse *cyclin B2* and mouse *Ube2c* promoters [8].

Using global expression profiling strategies many cell cycle regulatory genes have been shown to be regulated by the tumor suppressor protein p53. Induced by a myriad of different stress signals, which include amongst others DNA damage [15], hypoxia [16] and oncogenic stimuli [17], p53 protects the integrity of the genome either by inducing cell cycle arrest or provoking apoptosis. When activated, p53 mainly acts as a transcription factor. While cell cycle inhibitors (e.g. p21^WAF1/CIP1^) were transactivated by p53 [18], expression of many proteins supporting cell cycle progression and proliferation is shown to be repressed in a p53-dependent manner [19]. Various mechanisms are described for p53-dependent repression (reviewed in reference [20]), including direct DNA binding of p53 [21], interaction with other transcription cofactors [22] or with the basal transcription machinery [23], regulation of chromatin structure and promoter methylation [24], [25] or indirect mechanisms. The target gene *p21^WAF1/CIP1^* is of special importance for p53-dependent gene repression. It has been implicated in the repression mechanism of several genes. Furthermore, E2F binding sites in the promoters were implicated in this regulation [26]–[28]. Additionally, p21^WAF1/CIP1^ is sufficient to cause repression of those genes even in the absence of p53 [26]. However, most of these reports failed to identify E2F sites responsive to p53 and p21^WAF1/CIP1^. Interestingly, a large portion of genes down-regulated by p53 is also expressed in a cell cycle-dependent manner controlled by CDE/CHR elements in their promoters which were recently correlated to DREAM binding [7], [8]. Very recently, we could demonstrate that p53 can repress human and mouse *cyclin B2* genes indirectly through a conserved CHR element [29].

In this report, we identify the gene for the microtubule-dependent molecular motor protein KIF23 as a new p53 target. Reduced expression of both splice variants of *KIF23* is demonstrated on mRNA and protein level after wild-type p53 induction. Furthermore, we show that p53-dependent repression requires p21^WAF1/CIP1^ expression and an intact CHR element close to the transcription start site of the *KIF23* promoter. We reveal a cell cycle-dependent switch from DREAM to MMB complex binding to the CHR and demonstrate a p53-dependent accumulation of the DREAM complex at the *KIF23* promoter.

## Materials and Methods

### Cell Culture, Synchronization and Drug Treatment

Parental human colon carcinoma HCT116 cells and HCT116 cells with targeted deletions in both p53 (HCT116 *p53−/−*) or p21^WAF1/CIP1^ (HCT116 *p21−/−*) alleles [30] as well as tet-off inducible DLD-1 cell lines carrying p53wt (D53wt) or p53R175H (D53mut) transgenes [31] were generously provided by Bert Vogelstein. D53wt/mut cells were cultured as described previously [32]. Induced p53 protein expression is achieved by doxycycline removal. Cells were harvested 9 h after p53 induction.

HCT116 cells, NIH3T3 mouse fibroblasts (DSMZ, Braunschweig, Germany), HFF human foreskin fibroblasts (ATCC, Manassas, VA, USA) and T98G human glioblastoma cells (ATCC) were grown in DMEM (Lonza, Basel, Switzerland) supplemented with 10% fetal calf serum (FCS, Lonza) and penicillin/streptomycin and maintained at 37 °C and 10% CO_2_.

HFF, NIH3T3 and T98G cells were synchronized in G_0_ by serum deprivation for 48 h, 60 h and 72 h, respectively. Cells were restimulated for cell cycle re-entry with 20% FCS in DMEM and collected at given time points. Cell cycle distribution was checked by FACS analysis.

Doxorubicin was used at a final concentration of 0.2 µg/ml for 48 or 72 h. The Mdm-2 inhibitor nutlin-3 (Cayman Chemical, Ann Arbor, MI, USA; 10 mM in DMSO) was applied at a final concentration of 5 µM for 24 h. An appropriate amount of DMSO served as solvent control for nutlin-3 treatment.

### RNA and Protein Extraction, Real-time RT-PCR

Total RNA and protein were isolated using TRIzol Reagent (Invitrogen, Carlsbad, CA) following the manufacturer’s protocol. One-step real-time RT-PCR was performed as described previously [33] employing the LightCycler 2.0 instrument (Roche, Mannheim, Germany) using the QuantiTect SYBR Green RT-PCR kit (Qiagen, Hilden, Germany). The following primers were used for real-time RT-PCR to discriminate between the two splice variants of *KIF23*: hKIF23 variant 1_for, 5′-CCA TCA CCT GTG CCT CTT TC-3′; hKIF23 variant 1_rev, 5′-AAG AGT AAC CTG CCG CAA TG-3′; hKIF23 variant 2_for 5′-GTA GAG TGG CAG CCA AAC AGC-3′, hKIF23 variant 2_rev 5′-CTG ATC AGG TTG AAA GAG TAA AGG C-3′. Primers hKIF23 variant 1+2_for 5′-TGC TGC CAT GAA GTC AGC GAG AG-3′ and rev 5′-CCA GTG GGC GCA CCC TAC AG-3′ detect both splice variants. L7 was used as an endogenous control employing the primers L7_for 5′-GCA CTA TCA CAA GGA ATA TAG GCA G-3′ and L7_rev 5′-CCC ATG CAA TAT ATG GCT CTA C-3′.

### SDS PAGE and Immunoblot

SDS PAGE and western blot were performed following standard protocols [33] using antibodies against KIF23 (anti-MKLP-1 (24), sc-136473, Santa Cruz Biotech, Santa Cruz Biotechnology, CA, USA, 1∶200 dilution), p21^WAF1/CIP1^ (EA10, Calbiochem, Merck KGaA, Darmstadt, Germany, 1∶500), p53 (DO-1, Calbiochem, 1∶2000), GAPDH (G9545, Sigma, 1∶500) or β-actin (AC-15, Sigma, Hamburg, Germany, 1∶5000). For detection of DREAM/MMB complex components, the following antibodies were applied: E2F4 (C-20, Santa Cruz Biotechnology, 1∶750) and p130 (C-20, Santa Cruz Biotech., 1∶1000). The B-Myb LX015.1 monoclonal antibody was generously provided by Roger Watson [34] and the LIN37 polyclonal antibody was a kind gift from James A. DeCaprio [10]. Nfya was detected with Nfya (G-2) monoclonal antibody (Santa Cruz Biotech., 1∶2000). For stripping, the membranes were incubated for 30 minutes in Restore Western Blot Stripping Buffer (Thermo Scientific, Rockford, IL, USA).

### Plasmids and DNA Probes

The human *KIF23* promoter (nt −1153 to +1, relative to the translational start site) was amplified from human genomic DNA and cloned as KpnI/NcoI fragment into the pGL4.10 firefly-luciferase reporter-gene vector (Promega, Madison, WI, USA). Mutation of the CHR element from TTTGAA to TGCATA was introduced with the QuikChange site-directed mutagenesis kit (Agilent Technologies, Santa Clara, CA).

The human p53wt expression plasmid was produced by amplifying the insert of pCMV-p53wt (kindly provided by Bert Vogelstein, reference [35]) and cloning in pcDNA3.1HisC (Invitrogen, Carlsbad, CA, USA). The expression plasmid coding for p53mut R175H was generated by site-directed mutagenesis. Expression plasmids for human p21^WAF1/CIP1^, pCEP-p21wt and pCEP-p21mut, were generously provided by Bert Vogelstein [18].

DNA probes for affinity purification with a fragment of the human *KIF23* promoter (nt −296 to +1, relative to the translational start site) were obtained by PCR using a biotinylated primer for labeling the 3′-end (Invitrogen). As a negative control, a fragment of the mouse *Gapdhs* promoter (nt −169 to +12, relative to the transcription start site) was used. Sequences of primers can be obtained upon request.

### Transfections and Luciferase Reporter Gene Assays

Transfections of control siRNA (medium GC content, Invitrogen) and validated Stealth™ p53 siRNA (Invitrogen, VHS40367) at a final concentration of 100 nM were performed with DharmaFECT-1 transfection reagent (Dharmacon, Chicago, IL, USA) at a final dilution of 1∶1000 according to the manufacturer’s instructions [36]. Cells were harvested after 48 hours.

To measure p53- and p21^WAF1/CIP1^-dependent promoter activity with luciferase reporter assays, HCT116 cells were plated in 24-well plates (75,000 cells per well) and transfected by lipofection with FuGENE 6 (Promega) according to the manufacturer’s instructions. Cells were cultured overnight before cotransfection of 250 ng of promoter reporter plasmids along with 25 ng of constructs expressing wild-type or mutant p53 or p21^WAF1/CIP1^ proteins and 25 ng of Renilla luciferase plasmid (pGL4.70, Promega). After 24 h, cells were collected by lysing the cells with 100 µl passive lysis buffer (Promega) per well.

For measuring cell cycle-dependent promoter activity, NIH3T3 cells were plated in 12-well plates (19,000 cells per well). After 24 h, cells were transfected with pGL4 reporter constructs (transfection mixture per well: 0.2 µg promoter reporter plasmid (pGL4.10), 0.02 µg Renilla luciferase plasmid (pGL4.70), 1.5 µl GeneJuice (Merck, Darmstadt, Germany), 60 µl DMEM without FCS). 24 h after transfection, cells were synchronized by serum starvation in DMEM without FCS for 60 h. After restimulation with 20% FCS in DMEM, cells were collected at given time points by lysing the cells with 200 µl luciferase lysis buffer (Promega) per well and stored at −80°C.

Luciferase activity was measured with the Dual-Luciferase Reporter Assay system (Promega) following manufacturer’s recommendations. Relative light units (RLU) were calculated by normalizing firefly luciferase activity to Renilla luciferase activity [8]. pGL4.10 empty vector served as vector control.

### DNA-affinity Purification

DNA-affinity purification of protein complexes with density arrested and asynchronous growing NIH3T3 cells was performed as described before [8].

### Chromatin Immunoprecipitations (ChIPs)

T98G and HCT116 wild-type cells were cross-linked with 1% formaldehyde for 10 min at room temperature. ChIPs were performed as described earlier [8]. The following antibodies were used to precipitate DREAM complex components: E2F4 (C-20, Santa Cruz Biotech.), p130 (C-20, Santa Cruz Biotech.), B-Myb (N-19, Santa Cruz Biotech.), p53 (Ab-6, DO-1, Calbiochem), LIN9 (ab62329, Abcam, Cambridge, UK) and another LIN9 antibody was a kind gift from James A. DeCaprio [10]. A non-targeting rabbit antibody (IgG, Dako, Glostrup, Denmark) was used as a control for nonspecific signals. For all precipitations 1 to 2 µg of antibody and 20 to 35 µl of Protein G Dynabead suspension (Invitrogen) were used. Immunoprecipitated DNA was used as template for quantitative real-time PCR which was performed on an ABI 7300 qPCR System (Applied Biosystems, Forster City, CA) using QuantiTect SYBRGreen PCR Kit (Qiagen). All PCR results were normalized to input controls. The following primers were used for qPCR: hKIF23-for 5′-TAG GAG ACA AGC GCC ACT TC-3′; hKIF23-rev 5′-ACG CTA AGA GCC CAG GTG A-3′; hGAPDHS-for 5′-AGA CCA GCC TGA GCA AAA GA-3′; hGAPDHS-rev 5′-CTA GGC TGG AGT GCA GTG GT-3′, hp21-for 5′-CTG AGC CTC CCT CCA TCC-3′; hp21-rev 5′-GAG GTC TCC TGT CTC CTA CCA TC-3′.

### FACS Analysis

Cells were fixed for at least 12 h at 4°C in one volume of PBS with 1 mM EDTA and three volumes of absolute ethanol. DNA was stained with propidium iodide (Sigma) at a final concentration of 10 µg/ml in presence of RNase A (10 µg/ml). DNA content per cell was measured by flow cytometry on a LSR II instrument (Becton Dickinson, Franklin Lakes, NJ). Data analysis was performed with WinMDI 2.9 software.

## Results

### *KIF23* mRNA Level is Strongly Reduced after Ectopic Expression of Wild-type p53

**Figure 1:**
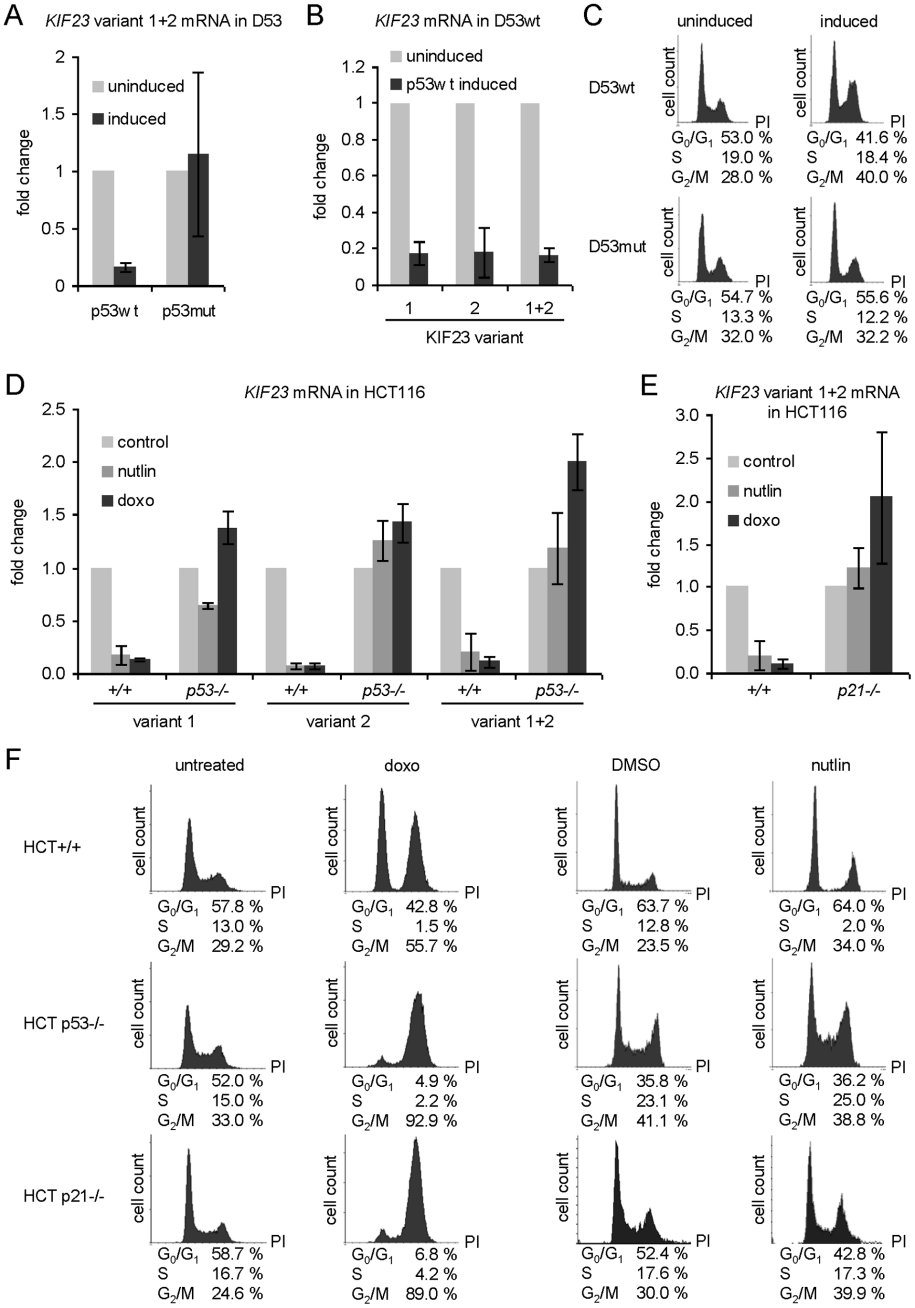
*KIF23* mRNA level is down-regulated by p53. *KIF23* mRNA levels were detected semi-quantitatively by real-time RT-PCR. Primers detecting both splice-variants of *KIF23* (variant 1+2) were employed as well as isoform-specific primers. Fold change of *KIF23* mRNA expression relative to not induced or untreated cells is displayed (mean ± SD, n = 3). (A) In the human colorectal adenocarcinoma cell line DLD-1 carrying tet-off inducible *p53* wild-type (p53wt) or R175H mutant (p53mut) transgenes in a functionally *p53*-negative background the expression of transgenes was induced for 9 h. (B) mRNA expression of *KIF23* transcript variants in D53wt cells after expression of p53wt was induced for 9 h. (D+E) HCT116 cells and HCT116 cells lacking (D) *p53* or (E) *p21* expression were treated with nutlin-3 and doxorubicin for 24 and 48 hours, respectively. DMSO- or untreated cells served as controls. (C+F) Cell cycle distribution of the cells was analyzed by FACS. In case of nutlin-3 treatment DMSO was used as solvent control.

In an indicator DNA microarray hybridization experiment, which had been performed earlier, using RNA from cells negative in functional endogenous p53 but stably transfected with a tet-off system for selective expression of wild-type p53 [37], it was observed that *KIF23* mRNA was 14.3-fold downregulated after ectopic p53wt expression (data not shown). In order to verify this observation we performed real-time RT-PCR analyses employing mRNA from the same cell line (D53wt) comparing *KIF23* mRNA levels before and after induction of wild-type p53 expression. We confirmed the data from the microarray experiment and found a strong decrease (6.3-fold) in *KIF23* mRNA expression level after wild-type p53 expression (Fig. 1A) which could not be observed in cells expressing mutant p53 deficient in DNA binding (D53mut) (Fig. 1A). As demonstrated by FACS analyses, cell cycle distribution was not altered significantly after induction of p53 mutant expression, but displayed enrichment in G_2_/M population after induction of wild-type p53 expression (Fig. 1C). This indicates that the expected upregulation of *KIF23* expression resulting from the shift in cell cycle distribution is overcompensated by p53-dependent repression.

Two splice variants are described for human *KIF23* [38]. Variant 1 represents the longer form and is also referred to as CHO1. It contains an additional exon (exon 18), which is missing in variant 2. So far, no differences in localization, expression pattern or regulation are known. As exon 18 is coding for an F-actin interacting domain [38], which may be essential for special functions of splice variant 1, we were wondering if mRNA expressions of the two splice variants of KIF23 were regulated differentially by p53. Employing specific primers to detect each splice variant of KIF23 separately, we found essentially the same p53-dependent downregulation of both mRNAs (Fig. 1B).

### Stabilization of Endogenous p53 Leads to a Decrease in *KIF23* mRNA Expression

In contrast to overexpression of p53 in the stably transfected DLD-1 cell line, *p53+/+* and *p53−/−* HCT116 human colon cancer cells provide an experimental system enabling inducible endogenous p53 expression at physiological levels. Comparison of gene regulation in HCT116 *p53+/+* cells with that in cells carrying targeted deletions in both p53 alleles (HCT116 *p53−/−*) allows direct testing of p53 function in an identical human cellular background [30]. We determined *KIF23* mRNA expression after stabilization of endogenous p53 in HCT116 cells treated with doxorubicin or nutlin-3. While the chemotherapeutic agent doxorubicin leads to stabilization of p53 protein through induction of posttranslational modifications of p53 by diverse kinases and acetylases [39], mechanism of nutlin-3 mediated p53-stabilization differs. Nutlin-3 inhibits interaction of p53 with Mdm2, impeding its ubiquitination and thereby proteasomal degradation. As shown in Fig. 1D, *KIF23* mRNA levels were drastically downregulated in HCT116 *p53+/+* cells after treatment with doxorubicin for 48 h (7.7-fold for splice variant 1 and 14,3-fold for splice variant 2, respectively) as well as after treatment with nutlin-3 for 24 h (5.6-fold for splice variant 1 and 14,3-fold for splice variant 2, respectively). Treatment of the isogenic cell line HCT116 *p53−/−* with nutlin-3 did not lead to a significant change in *KIF23* mRNA expression, while treatment of HCT116 *p53−/−* cells with doxorubicin led to a slight increase in *KIF23* mRNA expression (Fig. 1D). Analyses of each *KIF23* splice variant separately revealed nearly identical effects.

Remarkably, treatment of a further isogenic cell line with targeted disruptions in both *p21^WAF1/CIP1^* alleles (HCT116 *p21−/−*) with nutlin-3 or doxorubicin did not lead to a decrease in *KIF23* mRNA expression (Fig. 1E), even though two *p53* wild-type alleles were present. Instead, comparable with the effects in p53-negative HCT116 cells a 2.0-fold increase in *KIF23* mRNA expression after treatment with doxorubicin was observed. FACS data were obtained to rule out the possibility that *KIF23* expression is solely downregulated as an indirect effect caused by cell cycle arrest in G_1_. HCT116 cells show an increase in G_2_/M cell population after doxorubicin or nutlin-3 treatment whereas in no case the G_1_ cell population was enriched significantly (Fig. 1F). As expected, cell cycle distribution of HCT116 *p53−/−* cells after nutlin-3 treatment did not change considerably (Fig. 1F).

Taken together, these results demonstrate that *KIF23* mRNA expression is strongly repressed by wild-type p53. This effect can be observed in different cell lines and after different methods of p53 protein stabilization and seems to be dependent on p21^WAF1/CIP1^. Furthermore, p53-dependent repression is true for both *KIF23* splice variants.

### p53 Wild-type Reduces KIF23 Protein Expression

**Figure 2:**
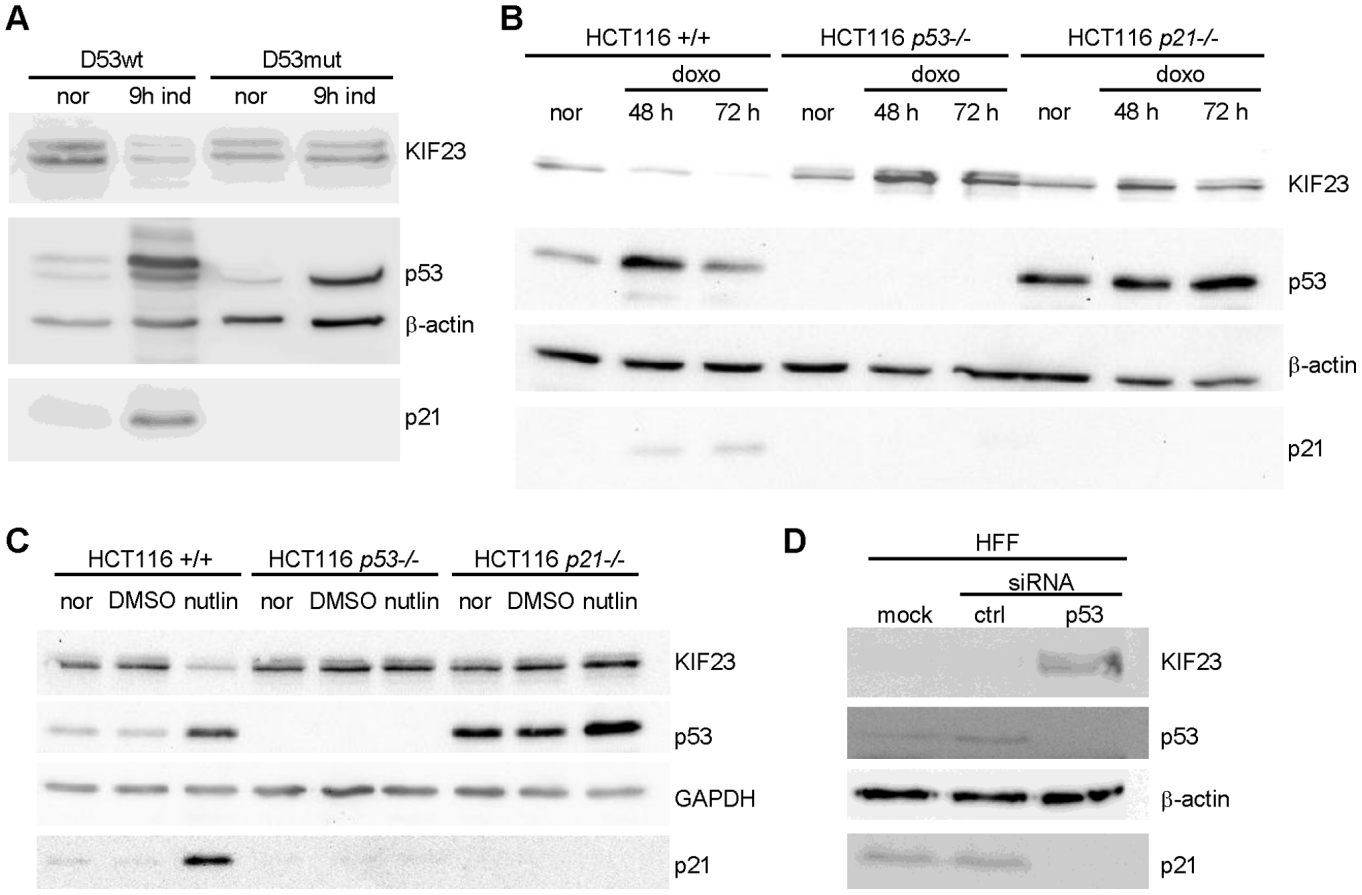
KIF23 protein levels are diminished after p53 activation and elevated following *p53* knockdown. KIF23, p53 and p21 protein levels were detected by western blot. GAPDH or β-actin served as loading control. (A) In D53wt or D53mut cells the expression of transgenes was induced for 9 h. (B+C) Wild-type HCT116 cells (HCT116*+/+*) or HCT116 cells lacking p53 (HCT116 *p53−/−*) or p21 (HCT116 *p21−/−*) were treated with (B) doxorubicin for 48 and 72 h or with (C) nutlin-3 for 24 h. DMSO was used as solvent control. (D) Human foreskin fibroblasts (HFF) were mock transfected or transfected with siRNA targeting p53 and control siRNA, respectively.

We next intended to examine effects of wild-type p53 expression on KIF23 protein levels. Analyses were carried out in both cell systems already tested for *KIF23* mRNA expression. Induction of ectopic p53wt expression in D53wt cells led to a significant decrease in KIF23 protein level while induction of DNA-binding deficient p53mut expression did not change KIF23 protein expression (Fig. 2A). Both splice variants of KIF23 are displayed as a double band in western blot experiments [40]. p53 and p21^WAF1/CIP1^ protein levels were analyzed as controls. As expected, both p53 and p21^WAF1/CIP^ protein expression levels increased 9 h after induction of ectopic wild-type p53 expression while no p21^WAF1/CIP1^ induction was observed in D53mut cells. β-actin is shown as a loading control (Fig. 2A).

Furthermore, western blot analyses of p53-positive HCT116 wild-type cells demonstrated an apparent loss of KIF23 protein expression after treatment with doxorubicin for 72 h (Fig. 2B) and a strong repression after treatment with nutlin-3 for 24 h (Fig. 2C). However, neither treatment of HCT116 *p53−/−* nor HCT116 *p21−/−* cells led to a decrease in KIF23 protein expression (Figs. 2B and 2C). Consistent with the results of the mRNA analyses, a slight increase in KIF23 protein expression could be observed after treatment with doxorubicin (Fig. 2B). Therefore, the results again indicate that p21^WAF1/CIP^ plays a critical role in the p53-dependent repression mechanism.

As elevated expression of p53wt led to a downregulation of KIF23 expression, we next asked if depletion of endogenous basal p53 reciprocally enhances KIF23 protein expression. To this end, we performed transient siRNA mediated knockdown of p53 in HFF cells. The results indicated that even basal levels of p53 are sufficient to suppress KIF23 expression in human foreskin fibroblasts. Depletion of endogenous p53 led to a clear increase in KIF23 protein levels, while expression of p21^WAF1/CIP1^ is abolished after treatment with p53-siRNA (Fig. 2D). As a control, neither KIF23 nor p21^WAF1/CIP1^ protein levels were affected by transfection of negative control siRNA.

### p53-dependent Downregulation of the *KIF23* Promoter Activity is Mediated through a CHR Element

**Figure 3:**
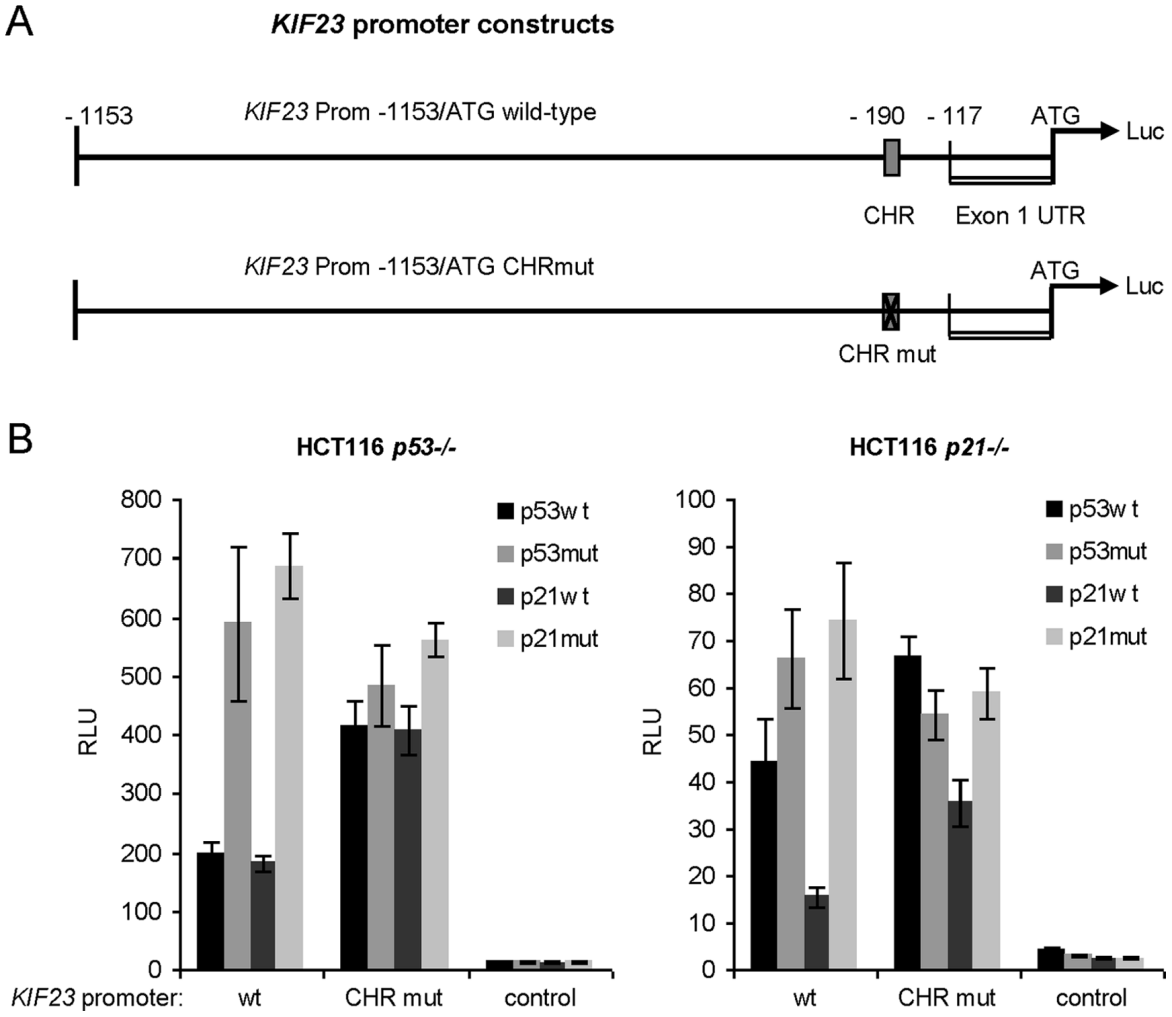
Repression of *KIF23* promoter activity by p53 depends on p21^WAF1/CIP1^ protein and the CHR element. (A) Schematic illustration of employed *KIF23* promoter constructs relative to the translational start site. (Luc = luciferase) (B) Dual luciferase reporter gene assays were carried out in HCT116 *p53−/−* (left) or HCT116 *p21−/−* (right) cells after cotransfection of p53wt, p53mut, p21wt or p21mut expression plasmids together with the *KIF23* wt promoter construct or a *KIF23* promoter construct with mutation of the putative CHR element. Empty firefly luciferase reporter vector served as vector control. Relative light units (RLU) are displayed.

In order to analyze the mechanism of p53-dependent repression of KIF23 expression we performed dual luciferase reporter gene assays employing the 1153bp *KIF23* promoter fragment upstream of the translational start site of the gene (KIF23 Prom -1153/ATG, Fig. 3A). Promoter activity was quantified in response to p53 or p21^WAF1/CIP1^ co-expression in HCT116 *p53−/−* and *p21−/−* cells. As indicated in Figure 3B, wild-type *KIF23* promoter activity was 3-fold downregulated by p53 in p53-negative cells. Remarkably, exclusive expression of p21^WAF1/CIP1^ in these cells also repressed *KIF23* promoter activity by 3.8-fold. In HCT116 *p21−/−* cells p21^WAF1/CIP1^ expression reduced *KIF23* promoter activity 4.7-fold, whereas p53wt expression only led to a 1.5-fold decrease in *KIF23* promoter activity (Fig. 3B).

In all cases, site-directed mutagenesis of the putative CHR element found 190 bp upstream of the translational start (Fig. 3A) abolished repression of *KIF23* promoter activity by p53 completely and left only a marginal CHR-independent repressive effect by p21^WAF1/CIP1^ (Fig. 3B).

As tested before, HCT116 cells transfected with p53 or p21^WAF1/CIP1^ expression plasmids showed only a slight increase in the G_1_ cell population and lowered fractions in G_2_/M phases. However, these small changes in cell cycle distribution are not sufficient to account for alterations in reporter activity [29].

On the basis of these data we conclude, that the CDK inhibitor p21^WAF1/CIP1^ seems to be both necessary and sufficient to reduce the promoter activity of the *KIF23* promoter effectively. Furthermore, we showed that the CHR element is necessary to mediate this repression.

### Expression of both Splice Variants of *KIF23* is Regulated Identically during Cell Cycle Progression and Requires a CHR in the Promoter

**Figure 4:**
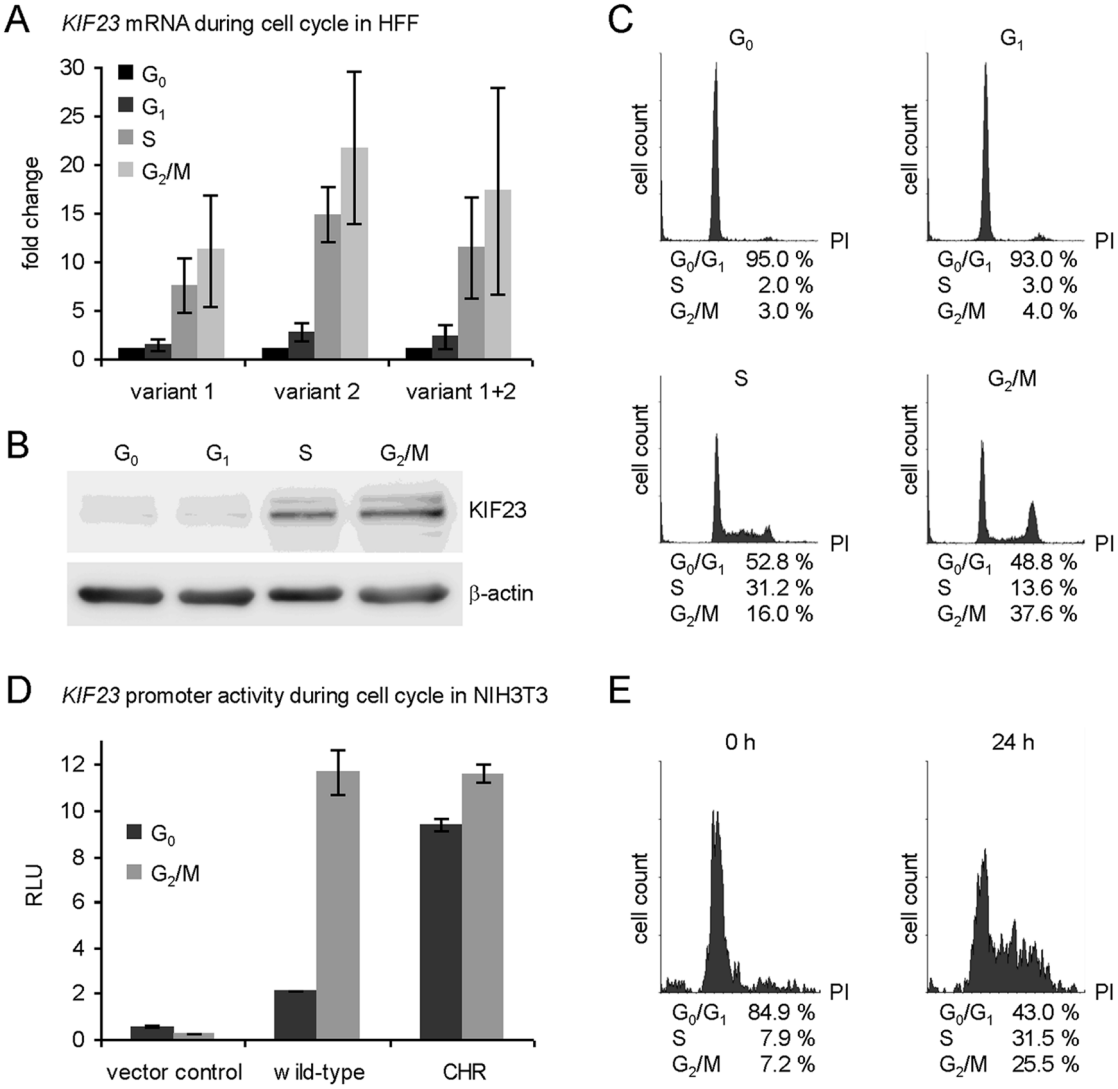
The CHR element mediates cell cycle-dependent expression of *KIF23*. Human foreskin fibroblasts (HFF) were arrested in G_0_ by serum starvation and re-entered the cell cycle after serum addition. Cells were harvested at 0 (G_0_), 12 (G_1_), 22 (S) or 26 (G_2_/M) hours after restimulation. (A) mRNA levels of *KIF23* splice variants were measured separately or with primers detecting both splice variants by real-time RT-PCR. (B) KIF23 protein levels were detected by western blot with β-actin serving as loading control. (C) Cell cycle distribution of HFF cells was confirmed by FACS analysis. (D) Dual luciferase reporter gene assays were carried out in serum-starved (G_0_) and 24 h restimulated (G_2_/M) NIH3T3 cells. Activities of wild-type and CHR mutant *KIF23* promoter constructs as well as the vector control are displayed as relative light units (RLU). (E) DNA content of serum-starved and 24 h restimulated NIH3T3 cells was analyzed by FACS.

Recently, Seguin and coworkers found expression of KIF23 to be regulated in a cell cycle-dependent manner in the human interleukin-2 (IL-2)-dependent Kit 225 T-cell line and in HCT116 cells [6]. We wanted to test for differences in cell cycle-dependent expression of both splice variants of KIF23. To analyze cell-cycle dependent expression, we employed normal human foreskin fibroblasts (HFF) as cell system, because they are non-transformed cells which can be easily starved by serum deprivation [41]. After starvation, HFF cells were restimulated to pass synchronously through the cell cycle. At different points of time after restimulation RNA and protein were extracted and analyzed. As displayed in Figures 4A and 4B both splice variants of KIF23 were regulated essentially synchronously in their mRNA (Fig. 4A) and protein (Fig. 4B) expression.

Consistently, promoter reporter assays demonstrated that *KIF23* promoter activity significantly increased from G_0_ to G_2_/M during the cell cycle. Mutation of the CHR element showed that cell cycle-dependent promoter activity strictly depends on the intact CHR (Fig. 4D).

### DREAM and MMB Complexes Bind to the CHR Element in the *KIF23* Promoter in vitro

The multi-protein complex DREAM has been shown to be responsible for repression of cell cycle-dependent genes in G_0_/G_1_ [10], whereas in proliferating cells recruitment of the MMB complex leads to increased expression of these genes [11], [13], [14]. Recently, Müller et al. showed that phylogenetically conserved CHR elements are enriched in promoters binding the DREAM complex. Specifically, DREAM binding to the CHR of *cyclin B2* and *Ube2c* promoters was demonstrated [8]. Thus, we wondered if these multi-protein complexes are also recruited to the CHR element of the *KIF23* promoter to regulate its cell cycle-dependent expression.

**Figure 5:**
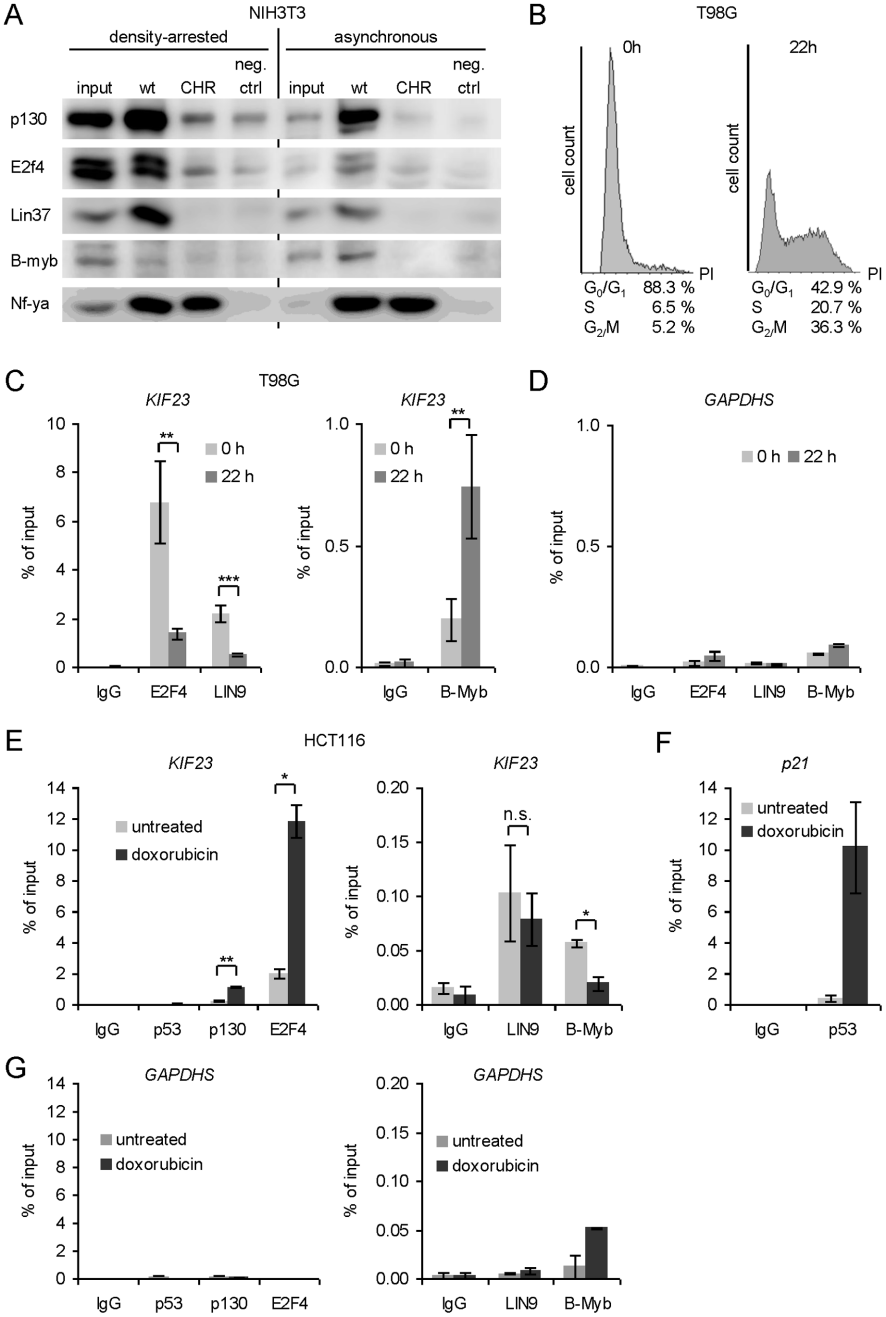
The DREAM and MMB complexes bind to the CHR in the *KIF23* promoter. (A) *In vitro* analysis of DREAM and MMB protein binding to the *KIF23* wild-type (wt) or the CHR mutant promoter. Nuclear extracts of density-arrested or asynchronously growing NIH3T3 cells were employed for DNA-affinity purification using biotinylated human *KIF23* promoter probes. Binding was tested by western blot analysis using antibodies against p130, E2f4, Lin37 and B-myb. As a protein binding to all *KIF23* probes, Nfya was detected. As a negative control, a fragment of the *Gapdhs* promoter was used. (B-D) T98G cells were synchronized by serum starvation and restimulated to re-enter the cell cycle for 22 h. (B) FACS analysis confirmed the cell cycle distribution. (C) Nuclear extracts were prepared and chromatin immunoprecipitation analysis of DREAM and MMB binding to the human *KIF23* promoter *in vivo* was performed with antibodies targeting E2F4, LIN9 and B-Myb. As a negative control, a non-targeting rabbit antibody (IgG) was used. All signals are given relative to the input (**p≤0.01; ***p≤0.001; n = 4). (D) The *GAPDHS* promoter served as negative control. (E-G) ChIP experiments were performed in untreated HCT116 cells or HCT116 cells treated with doxorubicin for 48 h. (E) Binding of p53, p130, E2F4, LIN9 and B-Myb to the *KIF23* promoter was analyzed (*p≤0.05; **p≤0.01; n.s. not significant; n ≥ 2) using (F) *p21* and (G) *GAPDHS* promoters as controls.

To this end, we performed DNA-affinity purification with biotinylated *KIF23* promoter probes and nuclear extracts of asynchronously growing NIH3T3 cells. Subsequent western blot analyses revealed that the DREAM components p130 and E2f4 as well as Lin37, a component of the MuvB core complex, and MMB complex component B-Myb were able to bind to the *Kif23* wild-type promoter *in vitro*. In density-arrested cells, a clear increase in protein expression of DREAM complex components p130 und E2F4 was observed and recruitment of these two proteins as well as Lin37 was increased seemingly. In contrast, MMB complex component B-myb seemed to be bound only in growing cells, as hardly any recruitment could be detected in arrested cells (Fig. 5A). Comparing binding of DREAM components to the CHR mutant of the *KIF23* promoter with nonspecific binding to the unrelated basal mouse *Gapdhs* promoter (negative control) revealed that DREAM recruitment to the *KIF23* promoter was reduced to background level after mutation of the CHR element. The nuclear transcription factor-Y subunit alpha (NFYA), which is able to bind to CCAAT-boxes [42] present in numerous cell cycle-regulated promoters including the *KIF23* promoter [6], served as a loading and binding control. As expected, binding of Nfya was observed independently of the CHR element (Fig. 5A).

These experiments demonstrate that the DREAM complex is recruited to the *KIF23* promoter in density-arrested cells *in vitro*, while the MMB complex only seems to bind to the *KIF23* promoter in growing cells. A functional CHR element is essential for both recruitments.

### *In vivo* Binding of DREAM and MMB Complexes to the *KIF23* Promoter is Cell Cycle-dependent

We assayed cell cycle-dependent binding of DREAM and MMB complexes to the *KIF23* promoter *in vivo* performing chromatin immunoprecipitations (ChIPs) in serum-starved (0 h) and restimulated (22 h) human glioblastoma T98G cells. The DREAM and MMB complexes have been well-characterized in this cell line [10], [11]. Cell cycle distribution of T98G cells was tested by FACS analyses illustrating cell cycle arrest in G_0_ after serum starvation and enrichment of cells in S and G_2_/M phases at 22 h hours after restimulation (Fig. 5B). ChIP experiments revealed that the DREAM component E2F4 was strongly bound to the *KIF23* promoter in G_0_-arrested cells (0h), supporting data from the DNA-affinity purification analyses, and was released from the *KIF23* promoter in restimulated T98G cells mainly in S/G_2_/M phases (Fig. 5C, left diagram). A small decrease in signal intensity was observed when analyzing promoter binding of LIN9, a component of the MuvB core complex. In contrast, an increase in B-Myb binding could be observed in growing cells *in vivo* (Fig. 5C, right diagram). No binding was detected analyzing the human *GAPDHS* promoter as negative control (Fig. 5D). Taken together, our results reveal binding of DREAM and MMB complex components to the *KIF23* promoter *in vitro* and *in vivo* in a cell cycle-dependent manner.

### p53-dependent Binding of DREAM/MMB Complex Components to the *KIF23* Promoter *in vivo*

Many genes whose expression is repressed by p53 are involved in cell-cycle control and contribute to p53-induced cell-cycle arrest. The promoters of a wide range of these target genes were found to bind DREAM and/or MMB-complexes in order to regulate their expression in a cell cycle-dependent manner [8], [10], [12]. We identified *KIF23* as a new p53 target gene and demonstrated that DREAM and MMB complexes bind to the CHR element in the *KIF23* promoter in a cell cycle-dependent manner. As we showed that this CHR is also responsible for p53-dependent regulation of the *KIF23* promoter, we next asked whether differential binding of DREAM and MMB may also occur in a p53-dependent manner. To this end, we performed ChIP assays using antibodies against DREAM complex components E2F4 and p130, MuvB core complex component LIN9 and MMB complex component B-Myb. Binding of these four proteins to the *KIF23* promoter in untreated HCT116 cells was compared with their promoter binding activities in cells treated with doxorubicin for 48 h to enrich endogenous p53 protein. Stabilization of endogenous wild-type p53 led to a clear increase in E2F4 and p130 binding (Fig. 5E, left diagram), whereas B-Myb binding significantly decreased (Fig. 5E, right diagram). LIN9 seemed to bind the *KIF23* promoter independently of p53 expression (Fig. 5E, right diagram). In contrast, no binding of p53 itself to the *KIF23* promoter was detected while a very strong binding of p53 to the *p21^WAF1/CIP1^* promoter could be demonstrated (Fig. 5F). The *GAPDHS* promoter served as negative control (Fig. 5G).

As shown in Fig. 1F doxorubicin treatment of HCT116 *p53+/+* cells increased the number of cells in G_2_/M. As a result, a shift from DREAM to MMB complex binding to the *KIF23* promoter would be expected. Instead, the observed increase in DREAM binding proved the effect of p53 on the *KIF23* promoter and showed that the observations are not only due to the overall changes in the cell cycle.

In summary, we show that *KIF23* gene expression is repressed by p53 and varies during cell cycle. We provide evidence that the DREAM complex binds to the CHR element of the *KIF23* promoter in a p53- and cell cycle-dependent manner. During cell cycle progression, DREAM binding is released when E2F4 and the pocket protein p130 is replaced by B-Myb to form the activating MMB complex in S/G_2_/M. In case of cellular stress, p53 seems to repress *KIF23* expression indirectly via induction of the CDK inhibitor p21^WAF1/CIP1^ and binding of the DREAM complex to the *KIF23* promoter.

## Discussion

Loss or mutation of p53 can lead to impairment of the centrosome duplication cycle, mitotic spindle abnormality and cytokinesis failure [43]. Assessing microarray data, Kinesin family member 23 (KIF23), which is essential for proper completion of cytokinesis, had been suggested as a potential p53 target gene earlier [19], [37]. However, no further investigations had been carried out to elucidate this correlation. Here, we demonstrated that p53 wild-type downregulates expression of *KIF23* at both mRNA and protein levels by regulation of *KIF23* promoter activity. This regulation of *KIF23* expression is consistent with the tumor suppressive function of p53, particularly as recent evidence indicates that *KIF23* could serve as a biomarker for some tumor entities. Among other regulators of cytokinesis, KIF23 expression was shown to be upregulated in non-small cell lung cancer and hepatocellular carcinoma [44], [45]. Very recently, expression analysis also revealed a higher level of KIF23 expression in glioma tissues compared to normal brain tissue and it was demonstrated that siRNA-mediated downregulation of *KIF23* expression significantly suppressed glioma cell proliferation *in vitro* [5].

Two splice variants of *KIF23* mRNA have been described previously [38] which differ from each other in the presence or absence of exon 18. The longer splice variant, also referred to as *CHO1*, contains exon 18, coding for an F-actin interacting domain. F-actin binding proteins have been shown earlier to be essential in cytokinesis [46]. However, no differential locations or activities have been described so far for the two *KIF23* splice variants and our data did not reveal any differential regulation of both slice variants either (Fig. 1B, 1D and 4A). For this, it has to be analyzed in further studies, whether there are different roles of KIF23 splice variants in human.

Several molecular mechanisms for p53-dependent repression of target genes, including direct and indirect mechanisms, have been described so far (reviewed in reference [20]). Some reports favour direct repression of G_2_/M genes through p53 [47], [48]. Others implicated the p53 target gene *p21^WAF1/CIP1^* and the pocket proteins p130 and p107 in p53-dependent repression mechanisms earlier [49]–[51]. The CDK inhibitor p21^WAF1/CIP1^ operates upstream of the RB/E2F pathway. By binding to both the cyclin and the CDK moieties, p21^WAF1/CIP1^ inhibits activities of all cyclin-CDK1/2 complexes as well as cyclin D-CDK4/6 complexes [52]. Thus, specific targets, including the pocket protein family (RB, p107 and p130), cannot be efficiently phosphorylated by the CDKs. For example, p130 is phosphorylated at serine 672 by CDK4/6 and subsequently targeted for proteasomal degradation [53]. Inhibition of cyclin-CDK activities by p21^WAF1/CIP1^ results in RB-family members remaining in a hypophosphorylated state bound to E2F transcription factors. Subsequently, accumulation of p130 stabilizes the DREAM complex [54], [55].

We showed, in concordance with genome-wide p53 binding studies [56]–[58], that p53 does not bind the *KIF23* promoter directly (Fig. 5E, left diagram). Furthermore, we demonstrated that p21^WAF1/CIP1^ is not only necessary but also sufficient to repress expression of *KIF23*. This strict p21^WAF1/CIP1^-dependency could be observed at both mRNA (Fig. 1E) and protein levels (Fig. 2B and 2C) after stabilization of endogenous wild-type p53 protein in the well-established cell line HCT116 *p21−/−*. In promoter reporter assays a minor p53-dependent and p21-independent repression of *KIF23* promoter activity was detected (Fig. 3B). This may be due to non-physiological high levels of p53 protein leading to an additional slight p21-independent repression of *KIF23* promoter activity. However, absence of p21^WAF1/CIP1^ led to a significant decrease in p53-dependent repression of *KIF23* promoter activity (Fig. 3B, right diagram) in this experiment as well, supporting the data of strict p21^WAF1/CIP1^-dependency of *KIF23* repression on mRNA and protein level after stabilization of endogenous wild-type p53 protein.

Very recently, it could be demonstrated that p53-dependent downregulation of *cyclin B2* expression, which also depends strictly on p21^WAF1/CIP1^ expression, is mediated by a switch of MMB to DREAM complex binding to a conserved CHR element [29]. Our current data reveal that nearly any repression effect of *KIF23* expression was lost after mutation of the CHR element in the *KIF23* gene (Fig. 3B). Different 5′-deletion mutants of the *KIF23* promoter construct did not lead to a significant change in p53-responsiveness (data not shown) although several E2F sites have been described for the *KIF23* promoter and a time-dependent recruitment of E2F1 to the *KIF23* promoter was observed [6]. Therefore we conclude that, instead of the E2F sites, the CHR is the major element mediating p53-dependent repression of *KIF23* expression. The quest for the proteins binding to CHR elements has taken very long. Very recently, Müller et al. found that the DREAM complex, consisting of the MuvB core, p130 and E2F4, can bind to the CHR in the *cyclin B2* promoter in resting cells to repress *cyclin B2* transcription, whereas the MMB complex, consisting of the MuvB core and B-Myb, appears to bind to the CHR element in proliferating cells to activate gene expression [8].

While the DREAM complex has been described as a global repressor of cell cycle genes, the MMB complex primarily targets many late cell cycle genes and activates their transcription in G_2_/M phases [8], [11], [12], [14], [59]. In this study we provide evidence for the first time that the DREAM complex is also able to bind to the CHR element in the *KIF23* promoter in resting cells *in vitro* (Fig. 5A) and *in vivo* (Fig. 5C). This binding occurs in a cell cycle-dependent manner with DREAM binding being substantially reduced in proliferating cells. In contrast, in resting cells the MMB complex hardly is observed at the CHR element of the *KIF23* promoter, but a clear DNA binding could be demonstrated in proliferating cells *in vitro* (Fig. 5A) and *in vivo* (Fig. 5C). Binding of both DREAM and MMB complexes is reduced to background level after mutation of the CHR in the *KIF23* promoter. As the CHR element was also found to be required for p53-dependent *KIF23* repression, we quantified DREAM/MMB complex binding depending on p53 wild-type expression. Interestingly, stabilization of endogenous p53 by treatment of the cells with doxorubicin seems to be followed by a switch from MMB to DREAM complex binding, with p130 and E2F4 being recruited while B-Myb was released from the complex (Fig. 5E).

Regarding the requirement of p21^WAF1/CIP1^ expression for proper regulation of *KIF23* transcription, we suggest the following molecular mechanism for p53-dependent repression: In absence of p53 only marginal amounts of the CDK inhibitor p21^WAF1/CIP1^ are expressed (Fig. 2B and 2C). Therefore cyclin-CDK complexes phosphorylate their substrates including B-Myb and the pocket protein family in a cell cycle-dependent manner [60], [61]. In G_0_/G_1_ hypophosphorylated p130 and E2F4 are bound to the MuvB core to form the DREAM complex [14] and transcription of cell cycle genes, including *KIF23*, is repressed. DREAM complex assembly is disrupted in early S phase by CDK4-dependent phosphorylation of p130 and E2F4 which impedes their binding to the MuvB core component LIN9 [14]. Simultaneously, association of the MuvB core with B-Myb is enabled by the cyclin A/CDK2-dependent phosphorylation of B-Myb [14]. Thereby, transcription of genes expressed in S phase and mitotis, including *KIF23*, is initiated [62].

In the presence of p53, induced expression of p21^WAF1/CIP1^ leads to inhibition of cyclin-CDK complexes so that p130, E2F4 and B-Myb remain hypophosphorylated. Comparable with the situation in resting cells, hypophosphorylated B-Myb is released from the MMB complex and the DREAM complex is assembled by recruiting hypophosphorylated p130 and E2F4 to the MuvB core [29], [54], [55]. Binding of the repressing DREAM complex to the CHR in the *KIF23* promoter finally leads to p53-dependent repression.

It is a remarkable finding that an identical molecular mechanism utilizing the same DNA element is responsible for cell cycle-dependent expression as well as for p53-dependent regulation of *KIF23* expression. Recent studies suggest that this molecular mechanism may be employed as a general mechanism for p53-dependent transcriptional repression of G_2_/M genes as for example *cyclin B2* and may contribute to carcinogenesis [8], [29], [54], [55]. DNA damage has been shown to result in a p53-dependent binding of p130 and E2F4 to and dissociation of B-Myb from the MuvB core [55]. Genome wide expression studies have shown that DREAM/MMB complexes are required for repression and activation of a cluster of genes essential for entry into mitosis, spindle assembly and cytokinesis [12], [13], [63], [64], most of which are bona fide p53 target genes. Recently, the CHR element was demonstrated to be the relevant DNA element responsible for DREAM/MMB binding to the promoters of a set of these genes [8]. This element is well known as the essential DNA site which confers cell cycle-dependent expression of G_2_/M genes [7].

Taken together our results suggest that the molecular mechanisms of both cell cycle-dependent expression and p53-dependent repression of molecular motor protein KIF23 are carried out by alternate binding of DREAM and MMB complexes to the CHR in its promoter. As many p53 target genes were expressed in a cell cycle-dependent manner, it can be assumed that this may be a common mechanism for transcriptional downregulation of cell cycle genes by the tumor suppressor protein p53. Further studies have to be performed in order to solidify this hypothesis. Clearly, detailed knowledge of molecular mechanisms of p53-dependent repression is important for the understanding of the process of tumor suppression and may lead to the development of novel anti-cancer therapeutics.
